# Supplementary material for: Molecular characterization of a Novel NAD+-dependent farnesol dehydrogenase SoFLDH gene involved in sesquiterpenoid synthases from Salvia officinalis
Source: PLoS One. 2022 Jun 3;17(6):e0269045. doi: 10.1371/journal.pone.0269045 (PMC9165828; doi:10.1371/journal.pone.0269045)
Supplement: S1 Fig — (DOCX) [file pone.0269045.s001.docx]

**S1 Fig**. Typical GC-MS mass spectrographs for terpenoids from leaf of *A. thaliana* plants

**Tetrapentacontane**

**Trans-elaidic acid**

**Palmitic acid, trimethylsilyl est**

**Palmitic acid**

**Oleic acid**

**Phytan**

**Cadinane**

**2-Methyldecane**

***AtWT***

**Thiourea, tetramethyl-**

***SoFLDH***

**Dimethylsiloxane pentamer**

**Levo-β-Elemene**

**Cis-Caryophyllene**

**Gamma-Muurolene**

**(-)-β-Bourbonene**

**Cis-Thujanol**

**Trans-Phytol**

**Farnesan**

**Dodecanoyl chloride**

**Heneicosane**

**Heptadecane, 8-methyl-**
