# Supplementary material for: Molecular characterization of a Novel NAD+-dependent farnesol dehydrogenase SoFLDH gene involved in sesquiterpenoid synthases from Salvia officinalis
Source: PLoS One. 2022 Jun 3;17(6):e0269045. doi: 10.1371/journal.pone.0269045 (PMC9165828; doi:10.1371/journal.pone.0269045)
Supplement: Attachment — Submitted filename: 5 Responses to reviewers 17-03-2022-Dr. MA.doc [file pone.0269045.s002.doc]

**Responses to reviewers**

Dear editor,

Thank you for your useful comments. We have been revised the manuscript **“Molecular characterization of a Novel NAD+-dependent farnesol dehydrogenase *SoFLDH* gene involved in sesquiterpenoid synthases from *Salvia officinalis*”** and make corrections required by the reviewers and the detailed information are listed below point by point:

**Responses to Reviewer #1:**

**Dear reviewer, thank you very much for your valuable comments. We have revised our MS based on your suggestion. Our responses to your comments are shown below. I hope that you are satisfied with our revision and/or explanation.**

1. **Comment:**

“1. Authors need to reconstruct phylogeny based on Maxmium likelihood analysis. Please see:

The BAHD Gene Family in Cacao (Theobroma cacao, Malvaceae): Genome-Wide Identification and Expression Analysis

Magnesium transporter Gene Family: Genome-Wide Identification and Characterization in The obroma cacao, Corchorus capsularis and Gossypium hirsutum of Family Malvaceae”

Answer: We appreciate your valuable suggestion and we already reconstruct our phylogeny tree based on the Maxmium likelihood. As you can see in Figure 1 (**Note: we already rearrangement all the figures numbers according all the reviewers comments**).

2. **Comment:**

“2. The authors did not mention about the transformation stability. The authors did not used Southern or Northern blotting analysis experiment. The primers used in PCR is also not mentioned. The authors need to justify the choice in the discussion. I think the data of the manuscript is good but these two point give very much limitation to the study.”

Answer: Thank you for your valuable comments and already adds new experimental for confirmed the transformation stability such as: Western blotting (WB) and Quantitative RT-PCR. As you can see in (Figure 6c and d) (**Note: we already rearrangement all the figures numbers according all the reviewers comments**).

**Responses to Reviewer #2:**

**Dear reviewer, thank you very much for your valuable comments. We have revised our MS based on your suggestion. Our responses to your comments are shown below. I hope that you are satisfied with our revision and/or explanation.**

1. **comment:**

“Add abstract in the main text. Also, add the key findings and take-home message in 1-2 sentences at the end of the abstract.”

Answer: We appreciate your valuable suggestion and we have added and modified the abstract part in our MS according to your suggestion.

2. **comment:**

“Line 48, please specify the growth conditions”

Answer: Thank you for your valuable comment and we already specify the growth conditions.

3. **comment:**

“Line 53, PROTPARAM is an online tool/website, not software.”

Answer: Thank you for your valuable comment and we corrected these mistakes

4. **comment:**

“Line 84-85, also add “following the manufacturer's instructions”.

Answer: We appreciate your valuable suggestion and we have modified this part in our MS according to your suggestion.

5. **comment:**

“One of the major issues is the presentation of the results and discussion. Combining results and discussion can be considered. However, the authors must divide the contents into subheadings for more clarity. Also, the current discussion is quite superficial and must be improved accordingly.”

Answer: We appreciate your valuable suggestion and we have modified the results and discussion part in our MS according to your suggestion. Also, we already divide the contents into subheadings for more clarity. (**Note: we already rearrangement all the figures numbers according all the reviewers comments**).

6. **comment:**

“The results regarding metabolites can be better explained, not just describe the data. Also, explain what does dataset suggests. Thus, I strongly suggest dividing the results into different subheadings and improving the results and discussion presentation.”

Answer: We appreciate your valuable suggestion and we have modified the results and discussion part in our MS according to your suggestion. (**Note: we already rearrangement all the figures numbers according all the reviewers comments**).

7. **comment:**

“There are spacing and spelling mistakes. The authors are requested to proofread the whole text.”

Answer: Thank you for your valuable comment and we corrected these mistakes.

8. **comment:**

“The English quality can further be improved.”

Answer: Thank you for your valuable comment and we already raise our manuscript English quality.

**Responses to Reviewer #3:**

**Dear reviewer, thank you very much for your valuable comments. We have revised our MS based on your suggestion. Our responses to your comments are shown below. I hope that you are satisfied with our revision and/or explanation**

1. **comment:**

“Line 63-64: please rewrite this sentence.”

Answer: Thank you for your valuable comment and we already rewrite this sentence.

2. **comment:**

“All gene names should be in italic format”

Answer: Thank you for your valuable comment and we corrected these mistakes.

3. **comment:**

“Line 90: "85°C for 5 second", it is 5 second or 5 min? I think it is 5 min. Please check it”.

Answer: Thank you for your valuable comment and you are correct it is 5 min, we already corrected these mistakes.

4. **comment:**

“Line 100-102: After 33 cycles, the final extension (10 min at 68°C) was applied. Please consider it again.”

Answer: Thank you for your valuable comment and you are correct it is 1.5 min at 68 °C, we already corrected these mistakes.

5. **comment:**

“Line 223, 229, 238, etc.: protein name should not be italic.”

Answer: Thank you for your valuable comment and we already corrected these mistakes.

6. **comment:**

“Line 253: these instated to this.”

Answer: Thank you for your valuable comment and we already corrected these mistakes.

7. **comment:**

“Line 253: after "by" provide the names of the authors”

Answer: Thank you for your valuable comment and we already add the names of the authors.

**Thank you very much for your attention and consideration.**

**Yours Sincerely,**

**Corresponding author**
